# Broiler meat fatty acids composition, lipid metabolism, and oxidative stability parameters as affected by cranberry leaves and walnut meal supplemented diets

**DOI:** 10.1038/s41598-022-25866-z

**Published:** 2022-12-14

**Authors:** Arabela Elena Untea, Raluca Paula Turcu, Mihaela Saracila, Petru Alexandru Vlaicu, Tatiana Dumitra Panaite, Alexandra Gabriela Oancea

**Affiliations:** 1 Feed and Food Quality Department, National Research and Development Institute for Biology and Animal Nutrition, Calea Bucharest, No. 1, 077015 Balotesti, Ilfov, Romania; 2 Nutrition Physiology Department, National Research and Development Institute for Biology and Animal Nutrition, Calea Bucharest, No. 1, 077015 Balotesti, Ilfov, Romania

**Keywords:** Fatty acids, Lipid peroxides

## Abstract

A randomized complete block with a 2 × 3 factorial arrangement was used to design a nutrition experiment conducted for the evaluation of the relation between walnut meal (WM—6% inclusion rate) and cranberry leaves (CL—1% and 2% inclusion rate) supplements and their effects on tissue lipid profile, lipid metabolism indices and oxidative stability of meat. Semi-intensive system conditions were simulated for 240 Ross 308 broilers and the animals were reared on permanent shave litter in boxes of 3 m^2^ (40 broilers / each group, housed in a single box). The current study results showed that the diets enriched in linolenic acid (LNA) (WM diets) led to broilers meat enriched in LNA, but the synthesis of long-chain omega-3 polyunsaturated fatty acids (LC n-3 PUFA) was stimulated when the diets were supplemented with a natural antioxidants source (CL diets). The CL diet also exhibited the most powerful effect in counteracting the oxidative processes of meat.

## Introduction

The n-3 and n-6 series of polyunsaturated fatty acids (PUFA) are essential for the human diet, together with their ratio (n-6/n-3) being a marker of lifestyle. Alpha-linolenic (C18:3n3, LNA), eicosapentaenoic (C20:5n3, EPA), and docosahexaenoic acids (C22:6n3, DHA) are the main three PUFA n-3 demanded in the human diet known as beneficial nutrients for human health^1^. Long-chain PUFA n-3 (LC-PUFA n-3), EPA, and DHA are associated with beneficial effects on cardiovascular and autoimmune disease, cancer, diabetes, and others^2^. Taking into account that terrestrial plants do not contain LC-PUFA^3^, animal-origin food represents a valuable source of these nutrients. Evolution steps and adaptation capacities led to different bioconversion rates of PUFA to LC-PUFA specific for each species. It seems that the LC-PUFA biosynthesizing ability respects the order of fish > birds > mammalians^4^. In this context, marine sources were put under great pressure but the poultry industry can be an alternative for human nutrition^5^. Different nutritional strategies were applied to obtain PUFA-enriched animal-origin food. PUFA meat enrichment through animal nutrition strategy was mostly based on diets supplemented with linseed (oil, seeds, or meal)^6–8^, fish oil^9^, camelina (oil, cakes, meal), and microalgae^10^. Using this strategy, meat products showed an enhancement of LNA concentrations, and a better n-6/n-3 ratio but LC-PUFA was not much positively influenced. Studies on the conversion efficiency of LNA to LC-PUFA in the human body showed a rate below 1%^11^, but the same observation is valid for most livestock species^12^. Apart from this, sensory and lipid peroxidation negative effects related to the acceptability of meat on market, have to be considered when the lipid profile of meat is improved by animal feeding. To counter these negative effects, dietary antioxidants are used in animal nutrition, the synthetic forms being popular for many years. Naturally occurring antioxidants become an interesting option with proven benefits on performance and endogenous antioxidant systems of animals^13^. Herbs and spices (clove, cinnamon, pepper, ginger, thyme, etc.) or by-products derived from the food industry (grapes, tomato, orange, olives, etc.) are the most common phyto-antioxidants studied in poultry nutrition^14–16^.

Walnut (*Juglans regia* L.) is considered a strategic plant for human nutrition and a valuable source of antioxidants, PUFA, LA, and LNA. The polyphenols content in walnuts depends largely on genetic factors and environmental growing conditions, the majority being those of the non-flavonoid type belonging to ellagitannins, or hydrolyzable tannins^17^, but also has small quantities of catechins and proanthocyanins monomers^18^. Up to 47% of the processed mass represents meal after oil extraction and depending on the method used the obtained walnut meal can become a natural source of n-3 PUFA and antioxidants for animal nutrition^19^. Recent studies have reported that walnut meal as a by-product^20,21^ is rich in many nutrients and is a promising dietary source that can contribute to the development of functional foods^22^. It has been shown^23^, that using walnut seed meal in broiler diets decreased feed intake, serum total cholesterol, and abdominal and muscle fat. Also, there was an improvement in n-3 PUFA content in breast and thigh meat without affecting their organoleptic properties and oxidative stability.

Cranberry (*Vaccinium oxycoccos* L.) is a valuable source of polyphenols, organic acids, anthocyanins, lipids, fiber, and nutritive minerals, therefore representing a rich source of bioactive compounds and phytonutrients for both human and animal diets^24^. Although cranberries have traditionally been used for medicinal purposes, recent advances in nutritional science have shown that all components of the fruit, as well as their by-products resulting from processing, can be used in animal nutrition. Cranberry leaves are a good source of microelements, especially Fe, Mo, Mn, Cu, and B, depending on several factors^25^ and represent a potential source of health-promoting compounds. Due to their high vitamin content, cranberry fruits and their products exert antimicrobial, antifungal, anticancer, and detoxifying properties. Oszmianski et al.^26^ conducted a comparative study between cranberry fruits and leaves to evaluate the bioactive potential of the leaves due to the limited existing studies. Research results indicated that cranberry leaves was not only a valuable source of selected antioxidants, but they also contained significantly more polyphenols than the fruit and fruit-based products, flavanols and flavan-3-ols being the major polyphenol group identified. Therefore, cranberry leaves can be considered a promising alternative to the use of natural bioactive compounds in animal feed to improve their quality.

This paper aims to elucidate the relationship between the effects of walnut meal (WM) and cranberry leaves (CL) on tissue lipid profile, lipid metabolism indices, and oxidative stability of broilers’ meat.

## Results

According to the fatty acids (FA) profile presented in Table 1, it can be noticed that the n-3 FA concentrations are approximately double in the 6% WM diets. LNA was the only n-3 FA presents in the profile of the feed and even though the sum of n-3 FA in the structure of nutritional supplements was similar, the differences in the sum of n-3 FA noticed in the feeds are explained by the different inclusion rates of plants in the diets.

**Table 1:** Fatty acids profile in diet’s structure and nutritional supplements.

Specification	WM 0%			WM 6%			CL	WM
%	CL 0%	CL 1%	CL 2%	CL 0%	CL 1%	CL 2%		
Σ SFA	13.06	13.07	13.13	12.55	12.58	12.34	31.24	10.90
Σ MUFA	27.99	27.56	27.29	26.80	26.45	26.65	27.90	17.58
Σ PUFA	58.79	59.22	59.58	60.53	60.96	60.89	40.34	71.33
Σ n-3	0.77	0.70	0.72	1.51	1.95	1.80	10.30	10.56
Σ n-6	58.02	58.52	58.86	59.02	59.02	59.09	31.73	60.77
n-6/n-3	75.46	83.43	81.43	39.05	30.28	32.91	3.08	5.75

*WM* Walnut meal, *CL* Cranberry leaves, *SFA *Saturated fatty acids, *MUFA *Monounsaturated fatty acids, *PUFA* Polyunsaturated fatty acids.

The lipid quality indices belonging to breast meat are presented in Table 2 and the lipid structure of tissues is detailed in Table 3. SFA belonging to experimental groups registered lower values compared to control, while all PUFA increased under studied feed additives influence (Table 2). MUFA concentrations decreased significantly for all experimental groups compared to C, of which only C24 registered higher values for the 2% CL-supplemented group. Regarding n-3 FA, both CL and WM supplements had a significant effect on increasing their concentrations, especially LNA (C18:3n3) being almost double that of other groups under WM influence. Although n-3 PUFA concentration was higher in the WM group, the synthesis of LC-FA did not meet this tendency. LA (C18:2n6) recorded for experimental groups higher concentrations than the C group, tendency respected for all n-6 FA.

**Table 2:** Effect of WM and CL on lipid quality indices based on fatty acids profile of breast meat.

Parameter	WM 0%			WM 6%			SEM	*P*		
%	CL 0%	CL 1%	CL 2%	CL 0%	CL 1%	CL 2%		WM	CL	WM x CL
Σ SFA	31.10^a^	29.53^b^	29.33^b^	29.33^b^	29.26^b^	28.63^b^	0.6803	0.0024	0.0031	0.0878
Σ MUFA	36.56^a^	35.22^b^	30.14^d^	35.20^b^	31.23^d^	33.49^c^	0.4657	0.0065	0.0001	0.0001
LC n-3	0.811^c^	0.877^bc^	1.181^a^	0.965^b^	1.088^a^	1.115^a^	0.0243	0.0001	0.0001	0.0001
Σ n-3	1.590^e^	1.754^d^	1.984^c^	2.140^b^	2.320^a^	2.257^ab^	0.0053	0.0001	0.0001	0.0001
LC n-6	3.492^ cd^	3.545^ cd^	5.607^a^	3.252^d^	4.354^b^	4.025^bc^	0.1560	0.0127	0.0001	0.0001
Σ n-6	30.31^e^	32.63^d^	37.96^a^	32.69^d^	36.47^b^	35.03^c^	0.4895	0.0001	0.0001	0.0001
Σ PUFA	31.99^d^	34.60^c^	40.07^a^	35.02^c^	38.97^a^	37.43^b^	0.5377	0.0001	0.0001	0.0001
n-6/n-3	19.07^a^	18.61^a^	19.17^a^	15.27^b^	15.73^b^	15.53^b^	0.3265	0.0001	0.6632	0.1279
n-3(Δ5 + Δ6 DI)	1.221^b^	1.220^b^	1.496^a^	1.020^c^	1.254^b^	1.555^a^	0.0020	0.0001	0.0001	0.0001
n-6 (Δ5 + Δ6 DI)	6.575^a^	5.170^b^	6.995^a^	4.729^c^	5.651^b^	4.772^c^	0.0701	0.0001	0.0001	0.0001
n-3 EI	1.391^b^	1.306^b^	2.018^a^	1.024^c^	0.979^c^	0.982^c^	0.0435	0.0001	0.7410	0.0010
n-6 EI	0.089^a^	0.088^a^	0.095^a^	0.090^a^	0.090^a^	0.074^b^	0.0001	0.9690	0.0001	0.8902
**The efficiency of precursor conversion into product**										
n-3 (C18)	0.421^b^	0.482^a^	0.394^b^	0.288^d^	0.299^d^	0.342^c^	0.0006	0.0001	0.0072	0.0001
n-3 (C20)	0.329^c^	0.356^c^	0.268^d^	0.448^b^	0.456^b^	0.507^a^	0.0005	0.0001	0.0738	0.0001
n-3 (C22)	0.509^b^	0.587^a^	0.410^bc^	0.418^bc^	0.362^c^	0.315^c^	0.0048	0.0001	0.0007	0.0413
DHA/LNA	0.211^b^	0.265^a^	0.203^b^	0.145^c^	0.111^c^	0.114^c^	0.0110	0.0001	0.0390	0.0012
EPA/LNA	0.468^ cd^	0.533^ab^	0.511^bc^	0.359^e^	0.433^d^	0.572^a^	0.0128	0.0001	0.0001	0.0001
n-6 (C18)	0.0011^ab^	0.0009^abc^	0.0008^bc^	0.0013^a^	0.0008^bc^	0.0006^c^	0.0000	0.6793	0.0001	0.1738
n-6 (C20)	1.518^b^	1.494^b^	1.681^a^	1.437^b^	1.502^b^	1.478^b^	0.0287	0.0004	0.0029	0.0036
n-6 (C22)	1.033^a^	0.928^b^	1.093^a^	0.894^b^	0.936^b^	0.852^b^	0.0222	0.0001	0.1760	0.0001
AA/LA	0.086^b^	0.075^bc^	0.122^a^	0.065^c^	0.085^bc^	0.079^bc^	0.0049	0.0001	0.0001	0.0001

Means within a row with no common superscript differ (*p* < 0.05).

*SFA *Saturated fatty acids, *MUFA *Monounsaturated fatty acids, *PUFA *Polyunsaturated fatty acids, *LC n-3* Long-chain n-3 fatty acids, *LC n-6* Long-chain n-6 fatty acids, n3(Δ5 + Δ6 DI) or n6(Δ5 + Δ6 DI) Desaturase index Δ5 + Δ6 for n-3 or n-6 fatty acids, n-3 EI or n-6 EI Elongase index for n-3 or n-6 fatty acids, LNA (C18:3n-3); EPA (C20:5n-3); DHA (C22:6n-3); LA (C18:2n-6); AA (C20:4n-6).

**Table 3:** Effect of WM and CL on fatty acids profile in breast samples.

Fatty acids	WM 0%			WM 6%			SEM	*P*		
%	CL 0%	CL 1%	CL 2%	CL 0%	CL 1%	CL 2%		WM	CL	WM x CL
C6:0	0.032^ab^	0.016^b^	0.019^ab^	0.036^ab^	0.049^a^	0.046^ab^	0.0003	0.9801	0.0012	0.1103
C8:0	0.031^ab^	0.015^c^	0.015^c^	0.032^ab^	0.043^a^	0.025^bc^	0.0001	0.0001	0.0109	0.0028
C10:0	0.026^bc^	0.024^c^	0.029^abc^	0.023^c^	0.034^a^	0.032^ab^	0.0000	0.0178	0.0018	0.0013
C12:0	0.015^c^	0.021^bc^	0.079^a^	0.015^c^	0.063^ab^	0.024^bc^	0.0007	0.6232	0.0053	0.0003
C14:0	0.479	0.457	0.466	0.451	0.481	0.444	0.0015	0.5148	0.6539	0.2096
C15:0	0.527^ab^	0.460^ab^	0.479^ab^	0.483^ab^	0.408^b^	0.590^a^	0.0058	0.8453	0.0091	0.0211
C16:0	21.29^a^	19.81^b^	18.03^c^	20.07^b^	18.68^c^	18.74^c^	0.3453	0.0094	0.0001	0.0004
C17:0	0.129^d^	0.163^c^	0.198^a^	0.152^c^	0.186^b^	0.178^b^	0.0000	0.0007	0.0001	0.0001
C18:0	8.110^bc^	8.105^bc^	9.472^a^	7.553^c^	8.670^ab^	7.825^c^	0.2157	0.0014	0.0006	0.0001
C20:0	0.177^c^	0.188^bc^	0.243^a^	0.174^c^	0.204^b^	0.185^bc^	0.0002	0.0016	0.0001	0.0001
C24:0	0.289^d^	0.273^d^	0.302^ cd^	0.342^c^	0.441^b^	0.540^a^	0.0009	0.0001	0.0001	0.0001
C14:1	0.091^a^	0.074^ab^	0.045^d^	0.077^ab^	0.052^ cd^	0.063^bc^	0.0001	0.0769	0.0001	0.0001
C15:1	0.162^b^	0.153^b^	0.099^c^	0.231^a^	0.202^ab^	0.213^a^	0.0008	0.0001	0.0047	0.0211
C16:1	3.167^a^	2.688^bc^	1.844^d^	2.766^b^	2.045^d^	2.509^c^	0.0152	0.0045	0.0001	0.0001
C17:1	0.119^c^	0.157^bc^	0.198^a^	0.152^bc^	0.180^ab^	0.163^ab^	0.0005	0.3801	0.0001	0.0017
C18:1n-9	32.18^a^	31.40^a^	26.51^d^	31.33^a^	27.95^c^	29.76^b^	0.5074	0.1478	0.0001	0.0001
C22: 1n-9	0.062^ab^	0.043^b^	0.081^a^	0.047^b^	0.075^a^	0.046^b^	0.0002	0.2253	0.3251	0.0001
C24:1n-9	0.782^b^	0.700^bc^	1.355^a^	0.597^c^	0.727^bc^	0.738^bc^	0.0095	0.0001	0.0001	0.0001
C18:3n-3	0.441^c^	0.454^c^	0.487^c^	0.836^a^	0.867^a^	0.751^b^	0.0014	0.0001	0.0358	0.0001
C18:4n-3	0.322^c^	0.423^a^	0.316^c^	0.339^bc^	0.365^abc^	0.392^ab^	0.0013	0.3397	0.0006	0.0004
C20:3n-3	0.422^bc^	0.431^b^	0.684^a^	0.369^c^	0.451^b^	0.418^bc^	0.0012	0.0001	0.0001	0.0001
C20:5n-3	0.206^e^	0.242^d^	0.249^d^	0.301^c^	0.372^b^	0.429^a^	0.0004	0.0001	0.0001	0.0001
C22:5n-3	0.090^b^	0.085^b^	0.148^a^	0.173^a^	0.169^a^	0.182^a^	0.0008	0.0001	0.0053	0.0637
C22:6n-3	0.093^b^	0.120^a^	0.099^ab^	0.122^a^	0.096^ab^	0.085^b^	0.0003	0.5932	0.0484	0.0013
C18:2n-6	26.81^d^	29.06^c^	32.33^a^	29.40^c^	32.09^ab^	30.98^b^	0.5631	0.0001	0.0001	0.0001
C18:3n-6	0.029^ab^	0.026^ab^	0.025^b^	0.037^a^	0.026^ab^	0.018^b^	0.0000	0.8520	0.0009	0.0469
C20:2n-6	0.222^ab^	0.240^ab^	0.140^b^	0.278^a^	0.274^a^	0.291^a^	0.0047	0.0014	0.2932	0.1021
C20:3n-6	0.483^c^	0.508^c^	0.764^a^	0.494^c^	0.578^b^	0.586^b^	0.0012	0.0086	0.0001	0.0001
C20:4n-6	2.317^bc^	2.188^bc^	3.916^a^	1.907^c^	2.721^b^	2.447^bc^	0.1225	0.0006	0.0001	0.0001
C22:2n-6	0.171^b^	0.232^ab^	0.235^a^	0.234^a^	0.286^a^	0.280^a^	0.0012	0.0001	0.0005	0.8372
C22:3n-6	0.109^d^	0.204^abc^	0.196^bc^	0.186^c^	0.273^a^	0.263^ab^	0.0017	0.0001	0.0001	0.9633
C22:4n-6	0.188^b^	0.174^b^	0.358^a^	0.152^b^	0.221^b^	0.158^b^	0.0017	0.0001	0.0001	0.0001
C18:2	0.088^c^	0.219^a^	0.125^bc^	0.196^ab^	0.182^ab^	0.149^abc^	0.0025	0.0648	0.0064	0.0047
Other FA	0.332	0.636	0.454	0.435	0.526	0.441	0.0385	0.9127	0.0563	0.4219

Means within a row with no common superscript differ (*p* < 0.05).

*WM* Walnut meal, *CL* Cranberry leaves.

The ratio between n-6 and n-3 FA significantly decreased for groups that included WM supplements proving its beneficial effect on the quality of lipids (Table 2). The presence of CL (2%) in broilers’ diets (individual or mixed) increased the desaturase index specific for n-3. The n-6 desaturase index significantly decreased for all experimental groups except in the 2% CL group. The elongation index for n-3 also increased under CL (2%) influence and decreased in WM groups, n-6 EI being uninfluenced by treatments. The efficiency of conversion from precursor to product revealed that for n-3, 1% CL stimulated the desaturation process on C18 and C22 chains and WM influenced C20 desaturation. Overall, the ratio DHA/LNA (final product/precursor) recorded the major value for CL (1%) group, while the WM-supplemented groups registered significantly decreased values compared to other groups. In the n-6 series, only CL (2%) stimulated the desaturation process for C20. WM supplements led to decreasing values of the conversion rate on the C22 series compared to the C group. Overall, the ratio AA/LA (final product/precursor) recorded the major value for the 2% CL group. WM supplements affected LC-PUFA synthesis.

The lipid quality indices belonging to the liver are presented in Table 4 and the lipid structure of tissues is detailed in Table 5. In liver tissue, C14 and C16 SFA decreased under 2% CL influence, while C17 and C23 increased compared to the C group (Table 4). All MUFA decreased for experimental groups with one exception (2% CL—C24:1n9). Similar to the breast profile, in liver samples, LNA registered almost double values for WM-supplemented groups, but the tendency was not respected for others n-3 FA. The LA concentrations also increased for experimental groups compared with control and the tendency also was not respected for all n-6 FA. The total concentrations of n-3 and LC n-3 registered higher values for WM-supplemented groups (Table 4). The n-6/n-3 ratio was significantly influenced by WM presence in diets, resulting in a significantly lower n-6/n-3 ratio, for groups supplemented with WM compared to those without WM in their diets. The lipid quality indices regarding desaturases and elongases activity were decreased compared with the C group with few exceptions: for 1% CL and WM (separate supplemented groups) the n-6 desaturase index was increased and for 1% CL, the n-3 elongase index recorded increased values compared to other groups.

**Table 4:** Effect of WM and CL on lipid quality indices based on fatty acids profile of liver.

Parameter	WM 0%			WM 6%			SEM	*P*		
%	CL 0%	CL 1%	CL 2%	CL 0%	CL 1%	CL 2%		WM	CL	WM x CL
Σ SFA	40.31^b^	41.29^a^	36.63^ cd^	40.18^b^	37.01^c^	36.29^d^	0.1199	0.0001	0.0001	0.0001
Σ MUFA	24.02^a^	22.26^b^	20.79^d^	22.45^b^	22.03^bc^	21.40^ cd^	0.1783	0.0107	0.0001	0.0001
LC n-3	1.458^de^	1.476^d^	1.341^e^	2.135^a^	1.648^c^	1.850^b^	0.0283	0.0001	0.0001	0.0001
Σ n-3	1.809^c^	1.788^c^	1.752^c^	2.644^a^	2.326^b^	2.453^b^	0.0581	0.0001	0.0001	0.0001
LC n-6	12.05^b^	11.90^b^	12.68^a^	11.82^b^	10.56^c^	12.08^b^	0.1381	0.0001	0.0001	0.0013
Σ n-6	33.66^e^	34.30^d^	40.76^a^	34.54^d^	38.44^c^	39.73^b^	0.0918	0.0001	0.0001	0.0001
Σ PUFA	35.47^e^	36.09^d^	42.52^a^	37.19^c^	40.77^b^	42.18^a^	0.1020	0.0001	0.0001	0.0001
n-6/n-3	18.61^b^	19.19^b^	23.27^a^	13.09^d^	16.54^c^	16.20^c^	0.2047	0.0001	0.0001	0.0001
n-3(Δ5 + Δ6 DI)	2.527^a^	2.239^a^	1.836^b^	1.838^b^	1.489^c^	1.579^c^	0.0843	0.0001	0.0001	0.0001
n-6 (Δ5 + Δ6 DI)	12.46^b^	14.77^a^	12.14^b^	13.11^a^	10.64^c^	12.82^b^	0.5007	0.0001	0.0001	0.0001
n-3 EI	3.281^b^	4.065^a^	1.902^c^	2.181^c^	1.262^d^	1.398^d^	0.0535	0.0001	0.0001	0.0001
n-6 EI	4.352^a^	3.243^b^	2.817^bc^	3.472^ab^	3.192^b^	2.070^c^	0.2196	0.0040	0.0001	0.1453
**The efficiency of precursor conversion into product**										
n-3 (C18)	0.308^ab^	0.346^a^	0.271^b^	0.184^c^	0.189^c^	0.170^c^	0.0101	0.0001	0.0003	0.0361
n-3 (C22)	0.674^a^	0.629^b^	0.594^ cd^	0.617^bc^	0.556^e^	0.578^de^	0.0071	0.0001	0.0001	0.0010
DHA/LNA	1.843^a^	1.996^a^	1.533^b^	1.831^a^	0.967^d^	1.331^c^	0.0436	0.0001	0.0001	0.0001
n-6 (C18)	0.009^c^	0.010^bc^	0.012^b^	0.010^bc^	0.010^bc^	0.015^a^	0.0006	0.0286	0.0001	0.0673
n-6 (C20)	1.672	1.677	1.708	1.709	1.702	0.710	0.0101	0.1560	0.1011	0.2379
AA/LA	0.462^a^	0.448^a^	0.372^b^	0.442^a^	0.315^c^	0.375^b^	0.0057	0.0001	0.0001	0.0001

Means within a row with no common superscript differ (*p* < 0.05).

*SFA *Saturated fatty acids, *MUFA *Monounsaturated fatty acids, *PUFA *polyunsaturated fatty acids, *LC n-3* Long chain n-3 fatty acids, *LC n-6* Long chain n-6 fatty acids; n3(Δ5 + Δ6 DI) or n6(Δ5 + Δ6 DI) Desaturase index Δ5 + Δ6 for n-3 or n-6 fatty acids*, n3 EI or n6 EI* elongase index for n-3 or n-6 fatty acids, LNA (C18:3n-3); EPA (C20:5n-3); DHA (C22:6n-3); LA (C18:2n-6); AA (C20:4n-6).

**Table 5:** Effect of WM and CL on fatty acids profile in liver samples.

Fatty acids	WM 0%			WM 6%			SEM	*P*		
%	CL 0%	CL 1%	CL 2%	CL 0%	CL 1%	CL 2%		WM	CL	WM x CL
C14:0	0.337^a^	0.332^a^	0.216^c^	0.342^a^	0.264^b^	0.203^c^	0.0020	0.0001	0.0001	0.0001
C15:0	0.057	0.050	0.055	0.052	0.049	0.046	0.0094	0.3932	0.7624	0.8229
C16:0	20.65^a^	20.41^a^	17.91^c^	20.56^a^	18.71^b^	17.78^c^	0.0455	0.0001	0.0001	0.0001
C17:0	0.065^d^	0.069^d^	0.134^b^	0.072^d^	0.122^c^	0.146^a^	0.0001	0.0001	0.0001	0.0001
C18:0	19.02^b^	20.22^a^	18.05^c^	18.89^b^	17.52^c^	17.87^c^	0.0952	0.0001	0.0001	0.0001
C20:0	0.032	0.012	0.022	0.031	0.037	0.010	0.0006	0.6038	0.3481	0.1968
C23:0	0.144^c^	0.186^bc^	0.235^ab^	0.232^ab^	0.274^a^	0.236^ab^	0.0015	0.0001	0.0094	0.0129
C14:1	0.032	0.030	0.037	0.030	0.033	0.027	0.0001	0.2229	0.9338	0.1311
C15:1	0.100	0.122	0.162	0.132	0.158	0.159	0.0018	0.1313	0.0515	0.4904
C16:1	1.444^ab^	1.164^ cd^	1.085^d^	1.487^a^	1.240^c^	1.367^b^	0.0023	0.0001	0.0001	0.0001
C17:1	0.170^bc^	0.187^b^	0.208^a^	0.165^c^	0.167^c^	0.172^bc^	0.0001	0.0001	0.0001	0.0030
C18:1n-9	20.98^a^	19.56^b^	17.80^c^	19.49^b^	19.33^b^	18.39^c^	0.2167	0.0214	0.0001	0.0001
C22: 1n-9	0.216^a^	0.144^c^	0.116^d^	0.154^c^	0.180^b^	0.141^ cd^	0.0002	0.9661	0.0001	0.0001
C24: 1n-9	1.115^bc^	1.078^ cd^	1.401^a^	1.012^de^	0.951^e^	1.169^b^	0.0017	0.0001	0.0001	0.0013
C18:3n-3	0.243^e^	0.204f.	0.299^d^	0.415^c^	0.550^a^	0.500^b^	0.0001	0.0001	0.0001	0.0001
C18:4n-3	0.108^ab^	0.108^ab^	0.112^ab^	0.094^b^	0.129^a^	0.103^ab^	0.0002	0.8453	0.0295	0.0195
C20:3n-3	0.795^b^	0.829^b^	0.569^d^	0.904^a^	0.693^c^	0.699^c^	0.0017	0.0199	0.0001	0.0001
C22:5n-3	0.216^d^	0.240^d^	0.314^c^	0.472^a^	0.424^b^	0486^a^	0.0005	0.0001	0.0001	0.0002
C22:6n-3	0.447^de^	0.407^e^	0.458^d^	0.759^a^	0.531^c^	0.666^b^	0.0008	0.0001	0.0001	0.0001
C18:2n-6	21.36^e^	22.14^d^	27.73^a^	22.46^c^	27.58^a^	27.21^b^	0.0275	0.0001	0.0001	0.0001
C18:3n-6	0.192^d^	0.221^ cd^	0.333^ab^	0.228^ cd^	0.266^bc^	0.403^a^	0.0016	0.0008	0.0001	0.5486
C20:2n-6	0.269	0.239	0.234	0.214	0.219	0.221	0.0011	0.0540	0.5503	0.2756
C20:3n-6	0.792^b^	0.671^d^	0.852^a^	0.756^c^	0.817^ab^	0.796^b^	0.0004	0.0110	0.0001	0.0001
C20:4n-6	9.861^a^	9.910^a^	10.326^a^	9.921^a^	8.679^b^	10.193^a^	0.0829	0.0001	0.0001	0.0001
C22:4n-6	1.133^b^	1.083^b^	1.273^a^	0.937^c^	0.850^d^	0.871^ cd^	0.0016	0.0001	0.0001	0.0001
C18:2	0.047^a^	0.034^ab^	0.019^b^	0.032^ab^	0.031^ab^	0.036^ab^	0.0002	0.9401	0.0950	0.0167
Other FA	0.157	0.326	0.031	0.141	0.150	0.088	0.0431	0.5219	0.1263	0.3849

Means within a row with no common superscript differ (*p* < 0.05).

*WM* Walnut meal, *CL* Cranberry leaves.

**Figure 1:**
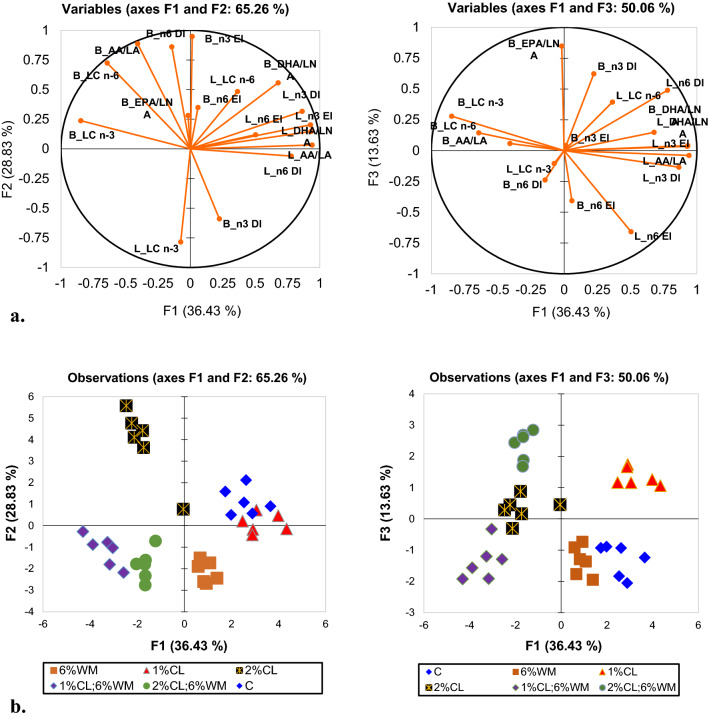
(**a**) The correlation circle represents the projection of considered variables in the factors space. (**b**) The distribution of the samples in the factor space. The variables considered were: elongation and desaturation indices for n-3 and n-6 FA series, long-chain FA n-3 and n-6 series, and efficiency of conversion: DHA/LNA, EPA/LNA, and AA/LA. The parameters notation was preceded by “L” or “B” corresponding to “liver” or “breast” tissue.

Principal component analysis (PCA) was conducted to explore the relationships between nutritional supplements used in the experiment and the FA metabolism parameters specific for liver and breast tissues. Figure 1a presents the correlation circle corresponding to the first three factors with eigenvalues > 1, which represent 78.89% of total variation (F1—36.43%; F2—28.83%; F3—13.63%). Figure 1b presents the distribution of the samples in the factor space. In the first diagram, F1 is related to the liver parameters and F2 is related to desaturase and elongase indices. Long-chain FA from the breast is negatively correlated with liver parameters but the conversion ratio of DHA from LNA is directly correlated with liver data. If the variables presented short vector length (like L_LC n-6; B_EPA/LNA or B_n-6 EI) the information might be better represented in other PCA like F1/F3. In the second diagram, it can be noticed that in breast n-3 and n-6 LC and AA/LA are very good correlated and negatively correlated with liver parameters. The distribution of the samples in the factor space showed that C and 1% CL were characterized by the well-correlated parameters of the liver and 2% CL was related to breast n-6 parameters (B_n-6 DI, B_LC n-6, B_AA/LA).

Data presented in Table 6 showed a positive influence of dietary supplements on lipid peroxidation parameters and myoglobin fractions. Primary oxidation products like peroxide value and conjugated dienes were decreased after 7 days of meat storage at refrigerated temperature when WM was included in diets, and the products formed in the latest stage of peroxidation were significantly decreased for all experimental groups. These achievements proved that the antioxidant compounds of vegetal materials included in the diets were very efficient in delaying the peroxidation process. Both phytogenic additives used in this experiment led to increased concentrations of Oxy Mb, by retarding the oxidation of iron from the second oxidation state to the third.

**Table 6:** Effect of WM and CL on oxidative stability of meat.

Parameter	WM 0%			WM 6%			SEM	*P*		
%	CL 0%	CL 1%	CL 2%	CL 0%	CL 1%	CL 2%		WM	CL	WM x CL
PV (Meq O2/kg)	0.514^ab^	0.510^ab^	0.532^a^	0.485^abc^	0.420^c^	0.438^c^	0.018	0.0001	0.2044	0.1656
CD (μmols/g)	25.61^a^	20.80^b^	21.19^b^	20.71^b^	19.03^b^	19.16^b^	0.717	0.0001	0.0002	0.0714
CT (μmols/g)	8.487	7.799	7.566	7.441	7.645	7.503	0.293	0.1152	0.1243	0.4061
P anis	58.69^a^	30.43^bcd^	40.37^b^	32.46^bc^	24.96^ cd^	21.13^d^	2.684	0.0001	0.0001	0.0010
TBARS (μg/kg)	351.03^a^	230.18^c^	264.47^bc^	292.77^b^	250.24^bc^	239.58^c^	11.66	0.0371	0.0001	0.0096
Met Mb (%)	61.15^a^	60.64^a^	60.50^a^	59.33^b^	59.86^b^	60.31^ab^	0.2877	0.0300	0.9354	0.2698
Deo Mb (%)	26.04	24.93	25.11	25.92	25.65	26.05	0.2970	0.0805	0.1527	0.2898
Oxy Mb (%)	13.03^b^	14.63^a^	14.66^a^	14.97^a^	14.73^a^	13.89^ab^	0.2624	0.1269	0.1374	0.0014

Means within a row with no common superscript differ (*p* < 0.05).

*PV* Peroxide value, *CD* Conjugated dienes, *CT* Conjugated trienes, *P anisidine* Para anisidine, *TBARS* Thiobarbituric acid reactive substances, *Met Mb* Metmyoglobin, *Deo Mb* Deoximyoglobin, *Oxi Mb *Oximyoglobin.

## Discussions

The manipulation of meat FA profile has been carried out using different sources of dietary PUFA. Also, some of the reported results proved that by using different nutritional supplements in animal diets, the essential FA from chicken meat can be transferred into human diets^3^. It seems that the PUFA metabolism of broilers is affected by dietary lipid sources and the bioconversion rate is limited by dietary supply^11^. The FAs with a higher number of carbon atoms called long-chain PUFA (LC-PUFA) are metabolically converted from LA and LNA through enzymatic pathways^27,28^. The same desaturase enzymes promote the desaturation of n-3 and n-6 C18 FA occurring in a permanent competition for the formation of LC-PUFA. By using diets enriched in LNA, it can be presumed that the transformation of LA into AA will be inhibited while EPA and DHA conversion will be stimulated.

The liver is the dominant player in the synthesis, oxidation, and deposition of FA^29^. The Δ5 and Δ6 are catalyzing enzymes (rate-limiting) that are responsible for LC-PUFA biosynthesis from their precursors^5^. These enzymes responsible for FA metabolism are expressed mainly in the liver and they are regulated by diet, hormones, and other factors^29^. As other reported results have shown, desaturase activity in non-hepatic tissues is very low, the liver is the fatty acids site production for peripheral tissues and in the present study, the desaturation and elongation activity were expressed by indices calculated as the ratio between the product and precursor ^30^. According to Gregory et al.^31^, a better understanding of LNA conversion into LC-PUFA n-3 is related to the status of elongase enzymes. It seems that ELOVL2 and ELOVL5 liver enzymes can elongate more efficient DPA to C24:5n3 in chickens compared to mammals and it led to a better synthesis and deposition of DHA.

In the current study, the most abundant FA in the lipid structure of the breast is oleic acid (C18:1n9) followed by linoleic (C18:2n6) and palmitic (C 16:0) acid, and in the lipid structure of the liver, palmitic (C16:0), stearic (C18:0), oleic (C18:1n-9) and linoleic (C18:2n-6) acids possessed similar ratios. The main n-3 LC-PUFA in breast meat and liver tissue was eicosatrienoic acid (C20:3n-3), a fatty acid produced by elongation from LNA. The bioconversion of LNA to stearidonic acid (18:4n-3) follows two metabolic pathways^32^: a desaturation step to C18:4n-3 and elongation to C20:4n-3 or elongation to C20:3n-3 and desaturation to C20:4n-3, the second pathway being a route not present in humans. Some researchers considered that the conversion of LNA to stearidonic acid is the rate-limiting step in the metabolism of LNA to DHA, but other studies proved that the elongation and desaturation steps involved in the conversion of DPA to DHA are also controlled points^33^.

For breast samples, in the control group, the conversion efficiency in the n-3 C20 carbon chain showed that WM-supplemented groups registered higher values. The minimum yield of conversion rate was noticed for the 2% CL supplemented group while in the next step of FA synthesis, the same group registered the higher value for the ratio between product and precursor (C22:5n-3: C20:5n-3), the elongation index presenting a maximum value for 2% CL group. All experimental groups presented significantly higher concentrations of EPA in breast tissues compared to the control group and all WM-supplemented groups and the 2% CL group had significantly higher concentrations of DPA. Finally, only groups supplemented with 1% CL and 6% WM individually, recorded higher concentrations of DHA in the breast (Table 3), with 1% CL recording the higher values for efficiency of conversion (DHA/LNA, EPA/LNA) (Table 2).

As in the case of n-3 PUFA, the LA (C18:2n-6) concentrations in the breast reflects the dietary n-6 supply, and all experimental groups recorded significantly increased concentrations compared to the control. The main pathway of LA (C18:2n-6) conversion to C20:3n-6 was by elongation to C20:2n-6 and desaturation to C20:3n-6. The highest concentrations of C20:3n-6 were noticed for 2% CL, WM + 1% CL and WM + 2% CL supplemented groups. The desaturation to arachidonic acid (AA) was most efficient for the 2% CL group. Arachidonic acid is the most important metabolite of LA and it was noticed that its concentrations are inversely proportional to n-3 LC-PUFA^34^. In the present study, the 1% CL and 6% WM individual inclusion groups recorded the higher DHA and the lowest AA concentrations.

Some authors suggested that a lower fat deposition in tissues is correlated with a higher rate of PUFA ingestion due to lipid metabolism pathways, like increased lipolysis or decreased lipogenesis and preferential oxidation of PUFA compared to SFA^35^. In birds, unlike mammals, fat from the tissues originates from liver lipid synthesis^36^. Compared to other tissues, the liver FA profile is not correlated with diet. The liver is the main site for de novo synthesis of FA but is not a storage organ^35^.

Jing et al.^29^ noticed that feeding diets with different LA: LNA ratios, the decreased values of the ratio are specific to the diets with an increased rate of n-3 PUFA incorporated in tissues. Our results showed that WM-supplemented diets had almost double LNA concentrations in the breast and liver and almost the same LA concentrations compared with other groups and in consequence, LA: LNA ratio had lower values than C or CL-supplemented diets. The competition between PUFA classes for the same desaturating and elongating enzymes may explain the effects observed, the most important values for desaturation and elongation index specific to the n-6 class of lipids in the breast, were recorded for the 2% CL supplemented group. As was expected, the conversion rate of LA to AA was decreased for WM groups compared to others, but the most important concentrations of AA were noticed for the 2% CL-supplemented group.

But the desaturase and elongase enzymes in breast samples led to a specific index for n-3 PUFA increased for 2% CL supplemented groups too. The enzymatic activity expressed as Δ5 + Δ6 desaturase index was more pronounced in 2% CL supplemented groups (without WM) for n-3 series accompanied by the major value for elongation index, proving that supplementation of broilers diets with 2% CL stimulated the synthesis of n-3 LC-PUFA in breast tissue.

The major values of efficiency of conversion for n-3 C18 and C22 were recorded for CL (1%) group. When the efficiency was calculated, the type of desaturase enzyme involved in the bioconversion process was not considered an influential factor. For example, in the C22 desaturation process, the Δ4 enzyme is involved^32^. The efficiency of bioconversion for n-3 (C22) recorded the major values for the 1% CL group and also the ratio DHA/LNA and EPA/LNA, calculated as an end product to precursor ratio, sustained the previous observations, CL (1%) being significantly different from other groups. Even if the desaturation index for n-3 and n-6 series indicated the most pronounced activity for the 2% CL group, the most efficient group for bioconversion of n-3 FA (DHA/LNA and EPA/LNA) was 1% CL. The efficiency of conversion specific only for n3 C20 showed a positive effect of WM in broilers’ diets. For n-6 conversion, 2% CL was the most efficient group.

A possible explanation for the facts presented before may be the increased endogenous oxidation process occurrence when n-3 PUFA-enriched diets are used. Research studies revealed significant changes occur in the expression of the oxidative stress response in the liver when PUFA diets are administrated. It was demonstrated the existence of a preferential order of oxidation (PUFA vs SFA) and the oxidation process is directed to PUFA provided in surplus by the diet. It was suggested that n-3 PUFAs surplus depress hepatic de novo lipogenesis and enhances FA oxidation capacity in the liver^37^. On the other hand, the presence of a dietary supplement that brings smaller quantities of n-3 PUFA, but is scientifically recognized as a powerful source of antioxidants, like CL, led to increased endogenous bioconversion of LC-PUFA from their precursors.

In Fig. 1a the considered variables were plotted as points in the factor space and they were represented by their correlations. The liver parameters (desaturase indices, LC-PUFA, elongase index for n-3 FA, and conversion efficiency of product to precursor) and breast parameters (DHA/LNA) are far from the center and close to each other, so they are positively correlated. All those parameters are negatively correlated with LC-PUFA n-3 from the breast, which is also far from the center but on the opposite side. These observations are confirmed by the squared cosines of the variables. On the vertical axis, breast n-6 parameters (DI, LC, and AA/LA) are negatively correlated with breast n-3 parameters (DI and LC). In the second diagram, it can be noticed that n-3 DI and EPA/LNA are correlated in breast tissue.

In Fig. 1b the observations are represented by their projections. According to the observation graphs and the squared cosines of the observations, the liver parameters are the dominant variables for C and 1% CL groups. The mixed group with 1% CL and 6% WM supplements is associated with LC-PUFA n-3. The group with 2% CL supplements was characterized by n-6 FA parameters of the breast (DI, LC, and AA/LA) while the dominant variable for WM single group is breast n-3 parameters (DI and LC). From the last diagram, we can conclude that the 2% CL plus 6% WM group is related to n-3 DI and EPA/LNA variables.

Based on statistical data and the relationships between liver and breast parameters it can be considered that the liver is the main production site for LC FA but the distribution and deposition of FA in peripheral tissues are not directly correlated with the production rate.

Fatty acid oxidation in meats is a process whereby PUFA reacts with reactive oxygen species (ROS) leading to a series of secondary reactions which in turn lead to the degradation of lipids. Dietary antioxidants can solve this problem by inhibiting the oxidation of double bonds in the PUFA chain via directly scavenging ROS or by delaying it via stalling the propagation phase^38^. In our study, the diet supplemented with WM had a powerful effect on primary oxidation products (PV and CD), decreasing their concentration, while the secondary products of oxidation (P-anisidine and TBARS) were reduced by the CL and WM supplementation. Taking into account the antioxidant potential of both supplements, a more pronounced effect was observed in delaying the oxidative process by decreasing levels of P-anisidine and TBARS in meat when both WM and CL were supplemented in the diet than these alone. The phenomenon of synergy between the antioxidant compounds contained in supplements may be the explanation for the protective effect of WM + CL against lipid peroxidation of meat. It was reported that some bioactive compounds express antioxidant effects in the initial phase of oxidation and others have an effect in the final phase of oxidation^8^, but no clear mechanism has been described. The effects observed may be attributed to important concentrations of polyphenols such as flavonols and flavan-3-ols^26^ from CL and tocopherol and catechins from WM^17^. Other authors found that cranberry leaves are also valuable sources of vitamin E, β-carotene, lutein, and zeaxanthin^39^ which may explain the radical scavenging activities with effect on the inhibition of lipid peroxidation.

Similar to lipids, proteins are attacked by ROS but also by lipid oxidation secondary products. The lipid oxidizing system includes some lipid oxidation products like MDA are ROS for protein oxidation^40^. Myoglobin is a heme protein from the muscle that determines the quality of meat. The relation between myoglobin and lipids oxidation can provide information regarding the efficiency of antioxidants present in diets on oxidative process delaying. Our findings showed a retard oxidation of MbFe^2+^ state (OxiMb) to Mb Fe^3+^ state (MetMb) in experimental groups. The WM groups provide increased concentrations of LC-PUFA, delayed lipid oxidation in the first and final stages, and also were effective in counteracting the oxidation of myoglobin. The presence of CL in broilers’ diets stimulated the LC-PUFA synthesis and also exhibited the main effect in delaying the oxidation process in meat, with a significant influence on myoglobin oxidation.

## Material and methods

The study was approved before the initiation of research, by the Ethical Committee of the National and Development Institute for Biology and Animal Nutrition (INCDBNA-IBNA), Balotesti according to experimental protocol no. 1375/ 2020 and in compliance with the Animal Research: Reporting of In Vivo Experiments (ARRIVE) guidelines. Also, the study complied with the principles of Romanian Law 43/2014 ordinance 28/31.08.2011 and Directive 2010/63/EU concerning the protection of animals used for scientific purposes.

### Experimental design

A randomized complete block with a 2 × 3 factorial arrangement was used to design a nutrition experiment conducted on mixed-sex broiler chicken aged 10 days. In the first 10 days (starter phase) all broilers received a conventional diet based on corn, wheat, and soybean meal, with 3008.00 kcal/kg metabolizable energy and 22% crude protein. Experimental diets were calculated according to the feeding requirements recommended by the National Research Council^41^ and the nutritional requirements of the Ross 308 hybrid^42^. Six dietary ratios which included three concentrations of cranberry leaves (0.1 and 2%) and two concentrations of walnut meal (0 and 6%) were developed as follows: (C) a control diet (corn-soybean diet with no nutritional supplements added); two experimental diets with 1 and 2% CL and no WM added; one experimental diet supplemented with 6% WM in CL absence and two mixed diets with 6%WM and 1 respective 2% CL (Table 7).

**Table 7:** Ingredients and chemical composition of broilers’ diets.

Diet composition (%)	Stage II grower (11–28 days)						Stage III finisher (29–42 days)					
	WM 0%			WM 6%			WM 0%			WM 6%		
	CL 0%	CL 1%	CL 2%	CL 0%	CL 1%	CL 2%	CL 0%	CL 1%	CL 2%	CL 0%	CL 1%	CL 2%
Maize	42.00	42.00	42.00	42.00	42.00	42.00	42.00	42.00	42.00	42.00	42.00	42.00
Wheat	13.38	16.77	14.82	15.91	13.97	12.00	20.56	18.60	16.65	17.75	15.79	13.84
Soybean meal	36.29	30.9	31.28	27.38	27.75	28.11	28.10	28.45	28.85	25.03	25.40	25.77
Vegetable (soybean) oil	3.49	4.68	5.25	3.96	4.54	5.12	5.11	5.67	6.26	4.97	5.55	6.12
Walnut meal	–	–	–	6.00	6.00	6.00	–	–	–	6.00	6.00	6.00
Cranberry leaves	–	1.00	2.00	–	1.00	2.00	–	1.00	2.00	–	1.00	2.00
DL- Methionine	0.28	0.25	0.25	0.30	0.30	0.31	0.20	0.20	0.20	0.25	0.26	0.26
Lysine	0.17	0.19	0.18	0.13	0.12	0.12	0.09	0.09	0.08	0.03	0.02	0.02
Threonine	0.02	0.03	0.03	0.10	0.10	0.10	0.10	0.10	0.10	0.07	0.07	0.07
Calcium carbonate	1.36	1.29	1.29	1.30	1.29	1.29	1.17	1.17	1.17	1.18	1.17	1.17
Calcium phosphate	1.61	1.49	1.50	1.52	1.53	1.54	1.30	1.35	1.32	1.35	1.36	1.37
Sodium chloride	0.36	0.36	0.36	0.36	0.36	0.37	0.33	0.33	0.33	0.33	0.34	0.34
Choline	0.04	0.04	0.04	0.04	0.04	0.04	0.04	0.04	0.04	0.04	0.04	0.04
Premix*	1.00	1.00	1.00	1.00	1.00	1.00	1.00	1.00	1.00	1.00	1.00	1.00
**Total**	100	100	100	100	100	100	100	100	100	100	100	100
*Calculated analysis*												
Dry matter	88.41	88.50	88.70	88.84	89.04	89.24	88.38	88.58	88.78	88.91	89.11	89.31
M.E. poultry, (kcal/kg)	3086.00	3086.00	3086.00	3086.00	3086.00	3086.00	3167.00	3167.00	3167.00	3167.00	3167.00	3167.00
Crude protein	20.00	20.00	20.00	20.00	20.00	20.00	19.00	19.00	19.00	19.00	19.00	19.00
Crude fat	6.47	6.51	7.08	6.68	7.25	7.83	6.92	7.49	7.46	7.67	8.24	8.81
Calcium	0.84	0.84	0.84	0.84	0.84	0.84	0.76	0.76	0.76	0.76	0.76	0.76
Available phosphorous	0.42	0.42	0.42	0.42	0.42	0.42	0.38	0.38	0.38	0.38	0.38	0.38
Lysine	1.19	1.19	1.19	1.19	1.19	1.19	1.05	1.05	1.05	1.05	1.05	1.05
Methionine	0.55	0.55	0.56	0.66	0.67	0.67	0.49	0.49	0.49	0.60	0.61	0.61
Met + cis	0.89	0.89	0.89	0.89	0.89	0.89	0.82	0.82	0.82	0.82	0.82	0.82
Threonine	0.78	0.78	0.78	0.78	0.78	0.78	0.71	0.71	0.71	0.71	0.71	0.71
Triphtopan	0.23	0.23	0.22	0.20	0.20	0.20	0.21	0.21	0.21	0.19	0.19	0.19

*1 kg of premix contains: 1,100,000 IU/kg vitamin A; 200,000 IU/kg vitamin D3; 2700 IU/kg vitamin E; 300 mg/kg vitamin K; 200 mg/kg Vit. B1; 400 mg/kg vitamin B2; 1485 mg/kg pantothenic acid; 2700 mg/kg nicotinic acid; 300 mg/kg vitamin B6; 4 mg/kg Vit. B7; 100 mg/kg vitamin B9; 1.8 mg/kg vitamin B12; 2000 mg/kg vitamin C; 8000 mg/kg manganese; 8000 mg/kg iron; 500 mg/kg copper; 6000 mg/kg zinc; 37 mg/kg cobalt; 152 mg/kg iodine; 18 mg/kg selenium.

Semi-intensive system conditions were simulated for 240 Ross 308 broilers and the animals were reared on permanent shave litter (10–12 cm thick) in boxes of 3 m^2^ (40 broilers / each group, housed in a single box), fed ad libitum, and free access to the water. The experiment lasted 32 days and environmental conditions were monitored throughout the experimental period. For the first 3 days of age, the room temperature was maintained at 33 °C and then gradually reduced to 22 °C, with an average temperature of 26.84 ± 3.45 °C throughout the experimental period. The light program was set according to the management breeding guide (23 h light / 1 h dark).

At the end of the experiment, on the last day of the finishing phase (42 days), 6 broilers/groups were randomly selected and slaughtered by cervical dislocation. Tissue samples (breast and liver) were collected for further analysis. The productive parameters (average daily gain, feed conversion) were calculated from the weekly records of the body weights and daily feed intake. For the study of oxidative parameters, breast samples were constituted and stored at refrigerated temperature (4 °C) for 7 days. After the storage period, oxidative stability parameters were performed.

### Chemical analysis

#### Fatty acids determination

The fatty acid content of the walnut meal, cranberry leaves, feeds and organic tissue samples (breast and liver) was performed using a Perkin Elmer Clarus 500 (Massachusetts, United States) gas chromatography. The method used aims to transform the fatty acids from the analyzed sample into methyl esters, separate the compounds on the chromatographic column, and identify them by reference to standard chromatograms. The gas chromatograph was equipped with a flame ionization detector (FID) and capillary separation column with high stationary polar phase TRACE TR-Fame, (Thermo Electron, Massachusetts, United States), measuring 60 m × 0.25 mm × 0.25 μm film^43^.

Lipid metabolism indices were calculated as follows:

The activities of desaturating and elongating enzymes converting PUFA in LC-PUFA were reported as indices, the efficiency of precursor conversion into product corresponding to each carbon chain length and the efficiency of precursor conversion into product expressed as DHA/LNA, EPA/LNA, and AA/LA, was calculated by relating the product to a precursor for each n-3 and n-6 fatty acids series. For a rigorous determination of the PUFA desaturation and elongation steps, it was considered the metabolic pathways described by^29^.

#### Oxidative stability parameters

Folch procedure modified was applied for the extractions of lipids. The minced breast sample reacts with a chloroform/methanol mixture (2:1, v/v). The homogenate was filtered in a separation funnel and the lower organic layer was collected, evaporated and stored for further analysis. The determination of peroxide value (PV) was performed by the ferric thiocyanate method, by dissolving the lipid extract in a chloroform/methanol (7:3, v/v) mixture. After the addition of xylenol orange (10 mmol/l) and FeCl2 (1000 mg/kg), the sample solution obtained was left at rest for 5 min and the absorbance was recorded at 560 nm. The value of conjugated dienes (CD) and trienes (CT) was determined by dissolving the lipid extract in 2,2,4-trimethylpentane (iso-octane) and recording the spectra at 233 nm (CD) and 268 nm (CT). The aldehydic compounds formed as peroxidation products were determined as p-anisidine values by measuring the absorbance at 350 nm of sample lipid solution dissolved in 2,2,4-trimethylpentane (iso-octane) before and after reaction with the p-anisidine reagent. The TBARS values (thiobarbituric acid reactive substances) were determined by the third derivative spectrophotometry modified method first described by^44^. The minced breast sample was treated with TCA (7.5%) /BHT (0.8%) mixture (2:1; v:v) and centrifuged at 3000 × g for 3 min. The filtered solution (2.5 mL) reacts with 1.5 mL 0.8% aqueous thiobarbituric acid solution at 80 °C for 50 min. The third spectra were recorded at 540 nm. The fractions of myoglobin were determined spectrophotometric according to the description of^45^ and the equation used for quantification was proposed by^40^.

The oxidative stability methods were previously described by Untea et al.^8^ and all determinations were performed using a UV VIS spectrophotometer (Jasco V-530, Japan Servo Co. Ltd., Japan).

### Statistics

The comparison for a randomized complete block design with a 2 × 3 factorial arrangement of treatments was performed using tested by two-way ANOVA (XLStat, Addinsoft, New York, USA) was used for performing statistical analysis) followed by Tukey's HSD test (*P* < 0.05). The statistical model included the fixed effects of WM (0 and 6%) and CL (0, 1% and 2%) and their interactions. Data were analyzed considering all broilers in a cage as an experimental unit.

To determine the relationships between lipid metabolisms parameters corresponding to each tissue sample considered, a principal component analysis (PCA) was performed using XLStat (Addinsoft, New York, USA).

### The chemical compounds studied in this article

Docosahexaenoic acid (PubChem CID:445580); Linolenic acid (PubChem CID:5280934); Linoleic acid (PubChem CID:5280450); Arachidonic acid (PubChem CID:444899); Eicosapentaenoic acid (PubChem CID:446284); Malondialdehyde (PubChem CID:10964); Thiobarbituric acid (PubChem CID:2723628).

## Conclusions

The current study results showed that the diets enriched in LNA (WM supplemented diets) led to animal origin food (broiler meat) enriched in LNA, but the synthesis of LC-PUFA n-3 was stimulated and the efficiency of conversion recorded maximum values when the diets were supplemented with a natural antioxidants source (CL supplemented diets) and also exhibited the most powerful effect in counteracting the oxidative processes occurred during the storage of meat.

## Data Availability

The datasets generated during and/or analyzed during the current study are available from the corresponding author on reasonable request.
